# Cognitive profiles in idiopathic normal pressure hydrocephalus and Alzheimer's disease

**DOI:** 10.1177/13872877261478264

**Published:** 2026-08-28

**Authors:** Magnhild S. Dejgaard, Per Kristian Eide, Gro Gujord Tangen, Eva Skovlund, Geir Selbæk, Torgeir Bruun Wyller

**Affiliations:** 1 Department of Geriatric Medicine, Oslo University Hospital, University of Oslo, Oslo, Norway; 2 Institute of Clinical Medicine, University of Oslo, Oslo, Norway; 3 Department of Neurosurgery, Oslo University Hospital, Oslo, Norway; 4 KG Jebsen Centre for Brain Fluid Research, University of Oslo, Oslo, Norway; 5 Norwegian National Center for Ageing and Health, Vestfold Hospital Trust, Tønsberg, Norway; 6 Department of Public Health and Nursing, Norwegian University of Science and Technology (NTNU), Trondheim, Norway

**Keywords:** Alzheimer's disease, Clinical Dementia Rating Scale, cognition, dementia, mild cognitive impairment, neuropsychological tests, normal pressure hydrocephalus, normative data

## Abstract

**Background:**

Idiopathic normal pressure hydrocephalus (iNPH) and Alzheimer's disease (AD) are neurodegenerative disorders with partly overlapping clinical features. Since the treatment is different, we need better knowledge about how the cognitive profile differs between these conditions.

**Objective:**

We aimed to compare the cognitive profile of iNPH patients with that of a large cohort of confirmed AD patients.

**Methods:**

Patients diagnosed with iNPH and accepted for shunt surgery were compared with patients with biomarker verified Alzheimer's disease in The Norwegian Register of Persons Assessed for Cognitive symptoms (NorCog). All patients underwent a standardized cognitive assessment with age and education adjusted z-scores. We used the Clinical Dementia Rating Scale (CDR) to adjust for disease severity. Since the cognitive score distributions were highly skewed, we used nonparametric analyses stratified by CDR stage combined with multinomial logistic regression.

**Results:**

In total, 276 iNPH patients were compared to 1113 AD patients. iNPH patients performed significantly poorer on phonemic fluency [median z-score difference (AD—iNPH) 0.30, 95% confidence interval (CI) 0.20 to 0.50], but significantly better on other cognitive tests, in particular immediate (median difference −0.35, 95% CI −0.49 to −0.20) and delayed recall (median difference −0.46, 95% CI −0.59 to −0.34). The differences persisted after adjustment for CDR and were most pronounced in early stages of the disease.

**Conclusions:**

iNPH seems to affect phonemic fluency more and memory less than AD. As the disease progresses, the cognitive profiles become more similar, and the conditions cannot be distinguished by cognitive tests.

## Introduction

Idiopathic normal pressure hydrocephalus (iNPH) and Alzheimer's disease (AD) are neurodegenerative diseases influencing cognitive function and gradually progressing over time. iNPH is characterized by one or more of the symptoms: gait disturbance, cognitive decline, and urinary incontinence.^1,2^ The pathophysiology is not fully understood, but disturbed cerebrospinal fluid (CSF) circulation is considered one of the contributing factors.^2–4^ The CSF disturbance can be treated by shunt surgery,^5^ with significant improvement in gait velocity and balance, whereas improvements in cognitive function are more uncertain.^6,7^ Differentiating iNPH from AD is challenging, due to overlapping symptoms and presumably coexisting neuropathological changes.^8,9^ According to American-European guidelines, a diagnosis of probable iNPH can be made if gait or balance disturbance is present along with either cognitive impairment and/or urinary incontinence.^1^ Both iNPH and AD are age-related, with a gradual onset and progressive worsening of symptoms.^10,11^ Since iNPH patients have more diverse symptoms in the early phase than AD patients, they might also seek medical attention earlier in the disease trajectory, thus confounding comparisons between the two patient groups. The cognitive profiles of iNPH and AD have been compared in small cohorts,^12–14^ and in early phases of disease, memory impairment is reported to be milder in iNPH than in AD.^14^ However, the lack of adjustment for possible differences in disease severity makes it difficult to interpret these findings. The prevalence of iNPH might be markedly higher than previously anticipated,^15–17^ with a gradual clinical deterioration and increased risk for death.^10,18^ Since iNPH is a treatable disorder and the outcome of treatment is dependent on early detection,^19^ we need a better understanding of clinical findings that can help in differentiating between the two diseases in an early phase. Accordingly, this study aimed to compare the cognitive profile between patients with iNPH and patients with AD, using age and education-adjusted z-scores, and adjusting for disease severity.

## Methods

This is a cross-sectional study comparing cognitive test profiles of patients with iNPH who were accepted for shunt surgery to those of patients with confirmed AD. We used age and education-adjusted z-scores and adjusted for the severity of cognitive impairment by the Clinical Dementia Rating Scale (CDR).

### Study population

iNPH patients were accepted for shunt surgery by the Department of Neurosurgery Rikshospitalet, Oslo University Hospital, between September 2018 and December 2023 and met the diagnostic criteria for possible iNPH according to the American European guidelines.^1^ Patients were selected for surgery based on MRI findings, supplemented with either measurements of intracranial pulse pressure (ICP) waves or MRI with intrathecal gadobutrol contrast (gMRI).^3,20^ Patient inclusion and clinical assessment have previously been described.^21^ The Alzheimer group consisted of patients included in The Norwegian Register of Persons Assessed for Cognitive symptoms (NorCog).^22^ NorCog is a national quality register that includes individuals assessed for cognitive symptoms in specialist health care in Norway and was established in 2007 to increase knowledge about cognitive impairment and dementia. So far, more than 27,000 participants have been included. Individuals included in NorCog undergo a comprehensive cognitive assessment, combined with brain imaging, blood samples and, for some patients, also CSF sampling. To ensure that the AD diagnoses were as accurate as possible, we selected patients who fulfilled the ICD 10 diagnostic criteria G30.0 or G30.1 and F00.0 or F00.1 and who also had a CSF analysis with a reduced concentration of amyloid-β (Aβ_42_) according to the reference limits at the time (see Supplemental Table 1).

### Assessment of cognitive function

All patients underwent a comprehensive cognitive assessment, and z-scores were calculated using age and education-adjusted norms. Z-scores indicate the number of standard deviations (SD) from the population mean, and a z-score of 0 thus corresponds to the average result for healthy individuals at the same age and educational level, a positive z-score indicates a performance above average, and a negative z-score indicates a performance below average. Whenever available, z-scores were based on Norwegian normative populations. The lowest possible z-score was minus 3.

A detailed description of the cognitive tests has previously been published.^21^ Briefly, we used immediate and delayed recall from either the Rey Auditory Verbal Learning Test (RAVLT) or the Consortium to Establish a Registry for Alzheimer's disease (CERAD) 10-word test.^23,24^ To be able to present a common measure of verbal memory, z-scores from both tests were calculated and merged into one common score for immediate and one for delayed recall. We used Espenes’ norms^25^ for RAVLT and Wagle's normative data^26^ for the CERAD-10-word test, except for individuals ≤ 69 years, where Kirsebom's norms were used.^27^ Visuoconstructive abilities were assessed using the Figure Construction Test from CERAD.^24^ Norwegian norms were not available, and z-scores were calculated based on population norms from Luck.^28^ To assess attention and psychomotor speed we used Trail Making Test (TMT) A and B. TMT B is also sensitive to executive function.^29^ For TMT A and B, we used Espenes’ norms.^30^ Individuals not able to complete these tests achieved a negative z-score of a magnitude dependent on the individually predicted scaled score based on their age and education as described by Espenes.^30^ Language abilities were tested using phonemic verbal fluency, which is also sensitive to executive function.^24^ For the phonemic verbal fluency tests, we calculated z-scores based on Egeland's Norwegian norms^31^ for patients ≤ 77 years. For those ≥ 78 years, Tombaugh norms were used since Norwegian norms were not available.^32^ Patients aged outside the age ranges for the respective normative populations were assigned z-scores as if they belonged to the youngest or the oldest age class, respectively, of the relevant population (see Supplemental Table 2). Z-scores were not available for all patients due to missing information about years of education (see Supplemental Table 3 for detailed information).

### Overall assessment of the severity of cognitive impairment

For a global assessment of cognitive impairment, we used the Clinical Dementia Rating Scale (CDR).^33^ This is a 5-point scale used to evaluate how cognitive impairment affects functional performance. CDR evaluates memory, orientation, judgement and problem solving, community affairs, home and hobbies, and personal care. These domain scores are combined based on an algorithm to generate a global score that reflects the severity of cognitive impairment, with greater weight given to memory items. A score of 0 signifies no cognitive impairment, 0.5 points mild cognitive impairment, 1-point mild dementia, 2-points moderate dementia, and 3-points indicates severe dementia. Due to the data distribution, CDR was merged into three groups: CDR 0–0.5 (no or mild cognitive impairment), CDR 1 (mild dementia), and CDR 2–3 (moderate or severe dementia).

### Statistical analysis

Since the cognitive score distributions were highly skewed, we used nonparametric methods throughout. We first estimated the median differences in each cognitive score between the AD group and the iNPH group by the Hodges-Lehmann estimator and tested the difference with the Wilcoxon Mann-Whitney (WMW) test. We then stratified the patients by CDR group (no or mild cognitive impairment, mild dementia, moderate or severe dementia) and estimated the median z-score differences for each cognitive test in each CDR stratum. Due to the highly skewed distributions, linear regression could not be used to model the z-scores as a function of patient group and CDR category. We explored the possibility to apply ordinal logistic regression, but for most of the variables, the assumption of proportional odds was not fulfilled. We thus carried out multinomial logistic regression analyses with patient group and three CDR strata as covariates and z-scores for the cognitive tests in the three ordered categories severe impairment (z ≤ - 3), moderate impairment (−3 < z ≤ -1.5) and no impairment (z > -1.5) as outcomes. This procedure gives two odds ratios (ORs) per cognitive test, one for severe versus no impairment and one for moderate versus no impairment. We did not perform additional adjustments for age, gender, or education, as these factors were already accounted for in the normative z-scores.

The CDR score was missing for a number of AD patients, and the stratified analyses as well as the multinomial logistic regression analyses could therefore only be carried out for a subset of the patients. To explore how this might impact the results, we carried out the following sensitivity analyses: 1) Stratified analyses as described above, but with patients missing the CDR score as a separate category. 2) Crude multinomial logistic regression analyses of the effect of patient groups upon the cognitive scores, carried out in the entire patient sample and not adjusted for CDR score. 3) Same crude analyses as in 2, restricted to the patients who also had a CDR score and thus took part in the main analyses. The statistical analyses were conducted using STATA/SE 18.0.

## Results

**Figure 1.**
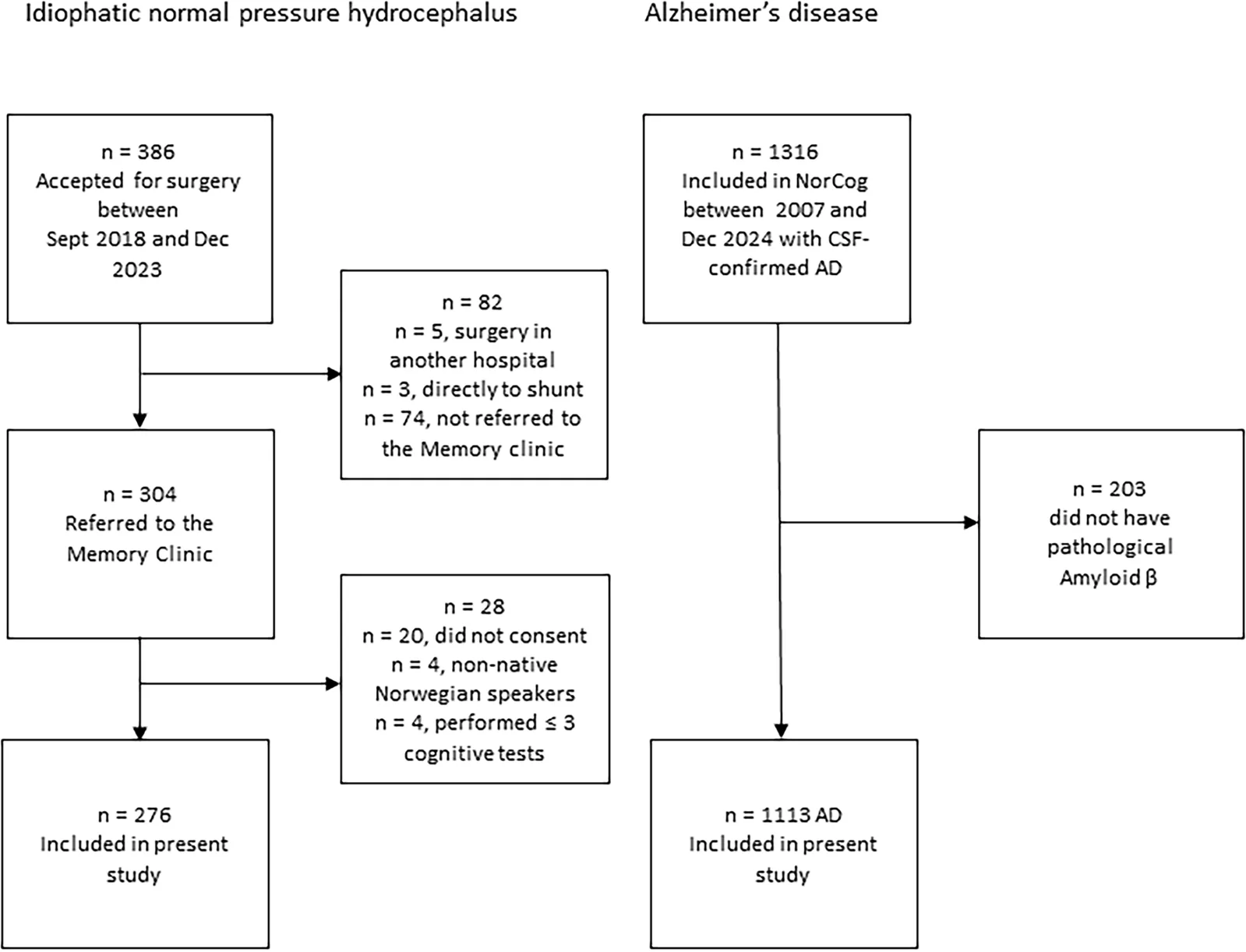
Flow chart for patients and reasons for dropout AD: Alzheimer's disease; NorCog: the Norwegian register of persons assessed for cognitive symptoms; CFS: cerebrospinal fluid.

In total, 276 iNPH patients were compared to 1113 AD patients. Flow chart and reasons for drop-out are presented in Figure 1. Demographic data and cognitive test results are described and compared between the two patient groups in Table 1. iNPH patients were older and had a higher proportion of men. There were no statistically significant differences in the length of education between the two groups. For both groups, most of the patients were in CDR group 0–0.5 (no or mild cognitive impairment) as presented in Table 2. In total, CDR was missing for 15 patients in the iNPH group and for 356 in the AD group.

**Table 1.** Demographic characteristics, cognitive tests scores and Clinical Dementia Rating Scores in patients with idiopathic normal pressure hydrocephalus (iNPH) and Alzheimer's disease (AD).

	Idiopathic normal pressure hydrocephalus			Alzheimer's disease			Median z-score differences, (95% CI)	p**
	n	**Mean (SD) or n (%)**		n	**Mean (SD) or n (%)**			
Age, years,	276	73.1 (5.7)		1113	70.0 (7.0)			<0.001
Male gender		167 (61%)			482 (43%)			<0.001
Education, years	272	12.5 (3.8)		1063	12.3 (3.6)			0.539

		**Median (IQR)**	**Median Z-score (IQR)**		**Median (IQR)**	**Median Z-score (IQR)**		
MMSE-NR, points (out of 30)	276	27 (24 to 29)		1080	22 (19 to 26)			<0.001
Clock Drawing Test, points (out of 5)	276	4 (3 to 5)		1041	3 (2 to 5)			<0.001
CERAD 10-word, immediate recall, words (out of 30)	77	11 (9 to 14)	−1.99 (−2.68 to −1.56)	944	10 (8 to 13)	−2.24 (−2.87 to −1.76)		
CERAD 10-word, delayed recall, words (out of 10)	77	1 (0 to 3)	−1.64 (−2.53 to −1.09)	929	1 (0 to 2)	−2.31 (−3.00 to −1.70)		
RAVLT, immediate recall, words (out of 75)	199	28 (22 to 35)	−1.77 (−2.51 to −0.96)	21	33 (29 to 38)	−2.20 (−2.80 to −0.69)		
RAVLT, delayed recall (trial 7), words (out of 15)	196	4 (2 to 6)	−1.85 (−2.43 to −1.15)	21	3 (2 to 6)	−2.57 (−3.00 to −1.74)		
Immediate recall ***	276		−1.86 (−2.55 to −1.07)	925		−2.24 (−2.83 to −1.73)	- 0.35 (−0.49 to −0.20)	<0.001
Delayed recall ***	273		−1.76 (−2.45 to −1.14)	911		−2.31 (−3.00 to −1.70)	- 0.46 (−0.59 to −0.34)	<0.001
CERAD Figure copying, points (out of 11)	264	10 (9 to11)	−0.50 (−2.10 to 0.40)	666	10 (8 to 11)	−1.10 (−3.00 to 0.40)	0.00 (−0.20 to 0.00)	0.022
TMT A, sec ****	269	63 (47 to 88)	−1.68 (−2.37 to −0.67)	1010	63 (45 to 91)	−1.94 (−2.64 to −0.70)	- 0.16 (−0.27 to 0.00)	0.028
TMT B, sec ****	180	161 (109 to 229)	−2.25(−2.60 to −1.34)	591	158 (109 to 241)	−2.37 (−2.81 to −1.35)	- 0.12 (−0.22 to −0.02)	0.009
Phonemic fluency, number of words	246	25 (17 to 31)	−1.50 (−2.30 to −0.80)	870	29 (20 to 38)	−1.20 (−2.00 to −0.30)	0.30 (0.20 to 0.50)	<0.001
CDR score	261	0.50 (0.50 to 1.00)		757	0.50 (0.50 to 1.00)			

Due to missing information about years of education, z-scores were not available for all patients included in the table. See Supplemental Table for detailed information.

*The Hodges-Lehmann estimator was used to analyze median z-score differences (AD-iNPH). **t-test for age, gender and education, Wilcoxon Mann-Whitney test for all other variables. ***For assessment of verbal memory, either CERAD 10-word test or RAVLT was applied. Z-scores were calculated for both, enabling a common measure for immediate and delayed recall across choices of verbal memory test. ****In the iNPH group, 4 participants were unable to complete TMT A and 93 were unable to complete TMT B. In the AD group, 18 participants were unable to complete TMT A and 208 were unable to complete TMT B. Mean, SD, median and IQR are for those who completed, whereas those who did not were assigned a negative z-score based on their age- and education-adjusted predicted score.. For details, see text.

IQR: interquartile range; MMSE-NR: Norwegian Revised Mini-Mental State Evaluation; CERAD: Consortium to Establish a registry for Alzheimer's Disease; RAVLT: Rey Auditory Verbal Learning Test; TMT: Trail Making Test; CDR: Clinical Dementia Rating Score.

**Table 2.** Z-scores for the cognitive tests stratified by Clinical Dementia Rating Scale (CDR) stage, with estimated median z-score differences.

	Idiopathic normal pressure hydrocephalus		Alzheimer's disease			
CDR	n	Median Z-score (IQR)	n	Median Z-score (IQR)	Median z-score differences, (95% CI) *	p**
**Immediate recall**						
0–0.5	189	−1.64 (−2.33 to −0.84)	384	−2.19 (−2.77 to −1.67)	−0.47 (−0.67 to −0.29)	<0.001
1	52	−2.13 (−2.82 to −1.55)	232	−2.35 (−3.00 to −1.78)	−0.15 (−0.47 to 0.00)	0.084
2–3	20	−2.64 (−2.93 to −1.88)	35	−3.00 (−3.00 to −2.15)	−0.16 (−0.40 to 0.00)	0.114
**Delayed Recall**						
0–0.5	187	−1.68 (−2.30 to −0.96)	381	−2.26 (−2.97 to −1.56)	−0.53 (−0.70 to −0.36)	<0.001
1	52	−2.09 (−2.71 to −1.39)	230	−2.32 (−3.00 to −1.63)	−0.17 (−0.40 to 0.00)	0.100
2–3	20	−2.17 (−3.00 to −1.61)	34	−2.77 (−3.00 to −1.94)	−0.04 (−0.73 to 0.09)	0.353
**Figure copying**						
0–0.5	185	0.20 (−1.40 to 0.40)	277	−0.50 (−2.50 to 0.40)	−0.05 (−0.20 to 0.00)	0.139
1	47	−1.50 (−3.00 to 0.20)	153	−2.30 (−3.00 to 0.30)	0.00 (−0.50 to 0.00)	0.576
2–3	18	−2.65 (−3.00 to −0.50)	18	−3.00 (−3.00 to 0.30)	0.00 (−0.50 to 0.40)	0.616
**TMT A**						
0–0.5	188	−1.23 (−2.29 to −0.37)	434	−1.41 (−2.48 to −0.46)	−0.16 (−0.37 to 0.00)	0.061
1	51	−2.27 (−2.53 to −1.73)	244	−2.37 (−2.70 to −1.15)	−0.00 (−0.16 to 0.16)	0.842
2–3	19	−2.48 (−2.70 to −2.10)	35	−2.48 (−2.75 to −2.06)	0.00 (−0.27 to 0.25)	0.863
**TMT B**						
0–0.5	188	−2.07 (−2.58 to −0.85)	370	−2.32 (−2.69 to −1.21)	−0.17 (−0.32 to −0.03)	0.013
1	51	−2.41 (−2.61 to −2.17)	157	−2.44 (−2.84 to −2.14)	−0.01 (−0.16 to 0.11)	0.767
2–3	19	−2.45 (−2.62 to −2.27)	20	−2.53 (−2.76 to −2.34)	−0.07 (−0.30 to 0.15)	0.482
**Phonemic fluency**						
0–0.5	175	−1.30 (−2.10 to −0.60)	363	−1.00 (−1.80 to −0.10)	0.30 (0.10 to 0.50)	0.003
1	43	−2.20 (−2.70 to −1.40)	201	−1.50 (−2.20 to −0.80)	0.50 (0.20 to 0.80)	0.001
2–3	15	−2.30 (−3.00 to −1.70)	32	−1.90 (−2.60 to −0.90)	0.50 (0.00 to 1.10)	0.068

CDR: Clinical Dementia Rating Scale; IQR: Interquartile range. TMT; Trail Making Test; WMW: Wilcoxon Mann-Whitney test.

*The Hodges-Lehmann estimator was used to analyze median z-score differences (AD–iNPH) **Wilcoxon Mann-Whitney test was used to test the differences in cognitive test results.

### Crude analyses

For phonemic fluency, the iNPH patients performed significantly poorer than the AD patients (median z-score difference 0.30 (95% CI 0.20 to 0.50)). For all other tests, the iNPH patients scored significantly better than the AD patients (Table 1). Memory was particularly reduced in the AD cohort, with a median z-score of −2.24 for immediate recall and −2.31 for delayed recall, compared to −1.86 and −1.76 in the iNPH group.

### Adjustment for the CDR category

**Figure 2.**
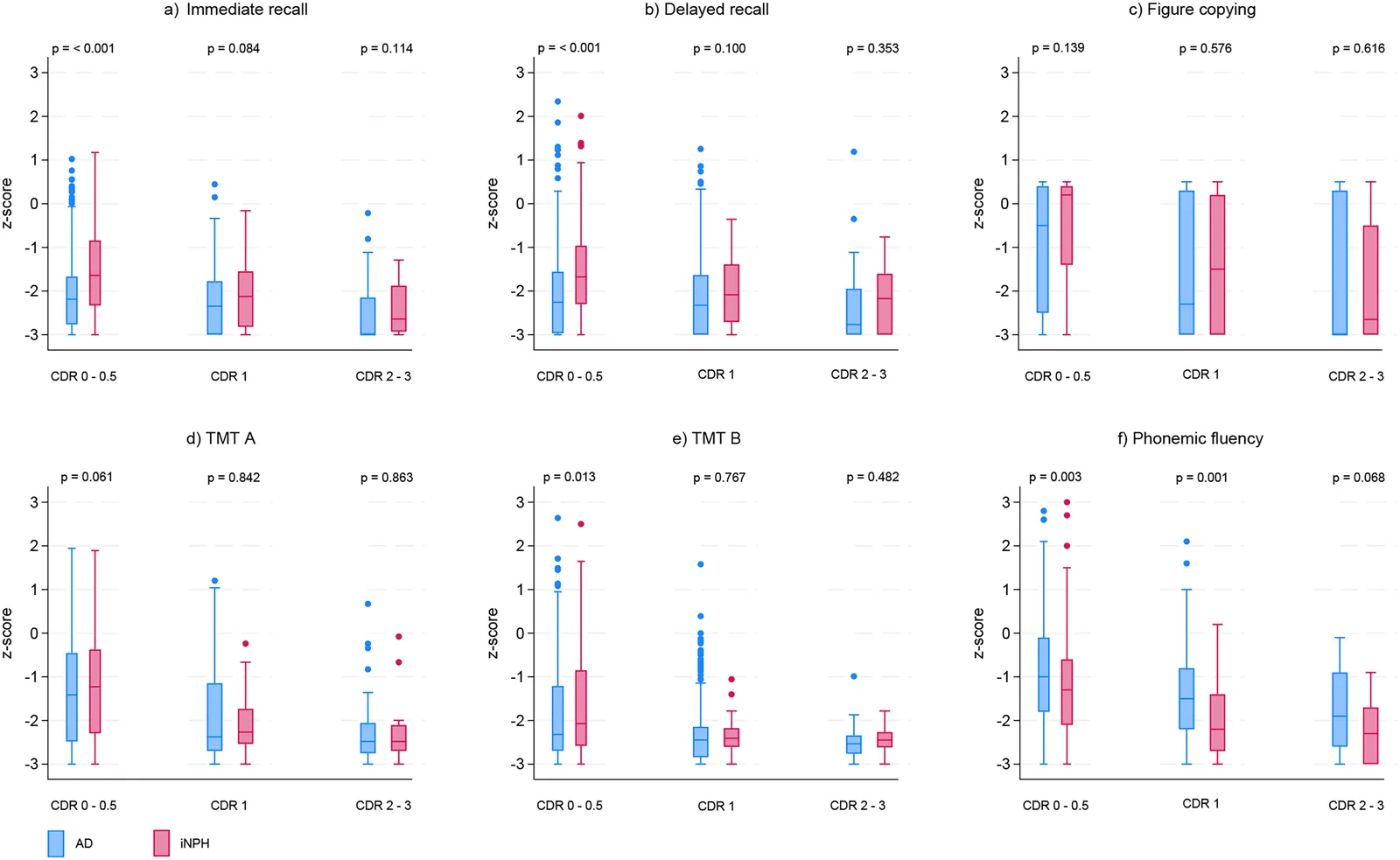
Cognitive test results in idiopathic normal pressure hydrocephalus and Alzheimer's disease, stratified by Clinical Dementia Rating scale (CDR) category TMT: Trail Making Test.

In Figure 2 and Table 2, the z-scores for the cognitive tests are compared between the iNPH and the AD group, stratified by CDR category (for those who had a CDR score). As expected, the median z-scores decreased with increasing severity of dementia for all the cognitive tests. As can be seen, the differences between the iNPH and the AD groups are in general most pronounced in the lower CDR categories (early phase of the disease) and are statistically significant only for immediate and delayed recall and TMT-B (in favor of the iNPH group) in the CDR 0–0.5 category and for phonemic fluency (in favor of the AD group) in the CDR 0–0.5 and CDR 1 categories. In the most severely impaired patients (CDR 2-3), no differences were statistically significant.

When adjusting for CDR category in multinomial logistic regression analyses (Supplemental Table 4, first column), all the differences in the cognitive scores remained statistically significant regarding the comparison severe versus no impairment, and for most of them also regarding the comparison moderate versus no impairment.

### Sensitivity analyses

Supplemental Figure 1 and Supplemental Table 5 show the same data as Figure 2 and Table 2, but with those missing the CDR score included as a separate category. As expected, this category is more heterogeneous, but the general tendency is the same as in the CDR-defined strata. In the second and third columns in Supplemental Table 4, the OR estimates are, in general, similar to those in the first column, indicating that the results seem valid even though several CDR scores are missing. An exception is the phonemic fluency test, for which the OR estimate for the comparison between severe and no impairment differs more between the models, indicating greater uncertainty regarding the impact of missing data in this analysis.

## Discussion

We found a notable difference in cognitive test profile; iNPH patients scored significantly better on nearly all cognitive tests, and particularly the memory tests, compared to AD patients. An important exception was phonemic fluency, for which the iNPH patients were more impaired. Our multivariable analyses, adjusting for CDR, indicate that the differences cannot be explained by systematic differences in diseases severity between the groups. The differences were more evident in early phase of the diseases, and the cognitive test profiles became more similar with increasing disease severity.

The phonemic fluency test is considered to be particularly sensitive for executive function.^31^ The cognitive profile in iNPH patients has sometimes been described as a fronto-subcortical pattern involving reduced executive function, slowed psychomotor speed and reduced memory.^12,34,35^ In a previously published study on the same iNPH cohort, we demonstrated that the patients scored below their age and educational expected norms for all the tests, without any particular pattern indicating a global cerebral dysfunction.^21^ Interestingly, when introducing a thoroughly diagnosed AD group as a comparator and adjusting for disease severity, it became evident that the iNPH patients were clearly more impaired regarding at least some elements of executive function, and less impaired regarding memory. The same pattern was found by Nerg and colleagues in a somewhat smaller sample.^14^ Executive functions are often associated with the frontal lobes and their connections,^36^ consistent with the assumption that ventricular enlargement in particular affects this region.

### Clinical implications

Cognitive tests alone cannot differentiate between iNPH and AD. An important finding in this study is that the distinction between the cognitive profiles of iNPH and AD is most pronounced during the early stages of cognitive impairment. As the diseases progress, the cognitive profiles seem to become more similar and more difficult to distinguish.

However, better knowledge regarding differences in their clinical characteristics helps select patients for further examination. It should indeed be assessed whether other elements of the iNPH triad (gait disturbance, urinary incontinence) are present, but a comprehensive mapping of cognitive problems may also be helpful. Many iNPH patients are probably misdiagnosed, and their symptoms are attributed to other neurodegenerative disorders.^37^ This is unfortunate because deterioration due to neurodegeneration cannot be regained through surgery.^10^ MR imaging markers for iNPH, such as increased Evans index, reduced callosal angle and dipropionate enlarged subarachnoid space hydrocephalus, are useful but nonspecific signs,^38^ and their diagnostic utility increases in combination with relevant clinical findings. Patients in whom clinical signs and initial imaging markers suggest iNPH should be referred to specialized centers for more definitive investigations to be carried out.

### Strengths and limitations

Strength in this study is the large patient population assessed by a standardized comprehensive test battery with available comparison to population norms. The iNPH group was compared with a clearly defined AD group confirmed by reduced Aβ_42_ concentrations in CSF. Included iNPH patients all fulfilled the American-European criteria for possible iNPH^1^ and were selected for surgery based on clinical assessment and MRI, complemented either with ICP wave monitoring or gMRI. Our hospital has a high success rate for surgical response to shunt surgery,^20^ indicating that the preoperative work-up is precise in identifying patients with shunt-responsive iNPH.

This study also has several limitations. Coexisting Alzheimer pathology is common in patients with iNPH,^8,9^ but was not assessed in our study. Including CSF markers or other AD biomarkers also in the iNPH group might have strengthened the results. Consequently, the study cannot determine how coexisting AD pathology influences the cognitive profile in iNPH patients or whether this profile differs between patients with iNPH alone and those with both iNPH and AD. On the other side, many patients with iNPH have more reduced levels of Aβ_42_ in CSF than can be explained by AD pathology,^39^ reducing the utility of this biomarker in the population at stake.

The NorCog Register currently comprises approximately 27,000 patients, of whom we included 1113. We thus included a highly selected group. This was, however, intended, as we wanted to compare the iNPH patients with patients who had a definite AD diagnosis, and the majority of patients in NorCog have not received an analysis of CSF biomarkers. The exclusion of clinically diagnosed AD patients may limit the extrapolation of the study results to the overall AD population.

Unfortunately, almost one in three AD patients did not have a CDR score registered. The fact that multivariate adjustment for CDR staging in general had a very weak impact on the estimates, and that the various sensitivity analyses changed the estimates minimally makes us confident, however, that the high percentage of missing CDR values does not have a major impact on the validity of our results. An unfortunate exception is the results regarding speech fluency, that were significantly affected by missing CDR scores. The CDR stratification made it obvious, however, that the difference in cognitive profile tends to be diluted as the diseases progress.

The study subjects were all Norwegian. Influenced by factors such as race, genetics, and healthcare environment, the extrapolation of the results to other ethnic and regional populations requires further validation. Further multi-center, cross-regional studies are needed to verify our findings.

Finally, this cross-sectional study could not assess how the iNPH patients’ cognitive characteristics change after shunt surgery. This must be assessed in separate studies.

### Conclusion

In the early phases of iNPH and AD, iNPH patients present with more severely impaired phonemic fluency, but better-preserved memory, than AD patients. The conditions cannot be distinguished by cognitive tests alone, but a cognitive test profile with particularly reduced phonemic fluency may help raise clinical alertness of iNPH. As the conditions become more severe, the test profiles become more similar. We recommend including at least one executive function test in clinical settings when patients are present with iNPH symptoms and particularly in early phase of the disease.

## Supplemental Material

sj-docx-1-alz-10.1177_13872877261478264 - Supplemental material for Cognitive profiles in idiopathic normal pressure hydrocephalus and Alzheimer's disease Supplemental material, sj-docx-1-alz-10.1177_13872877261478264 for Cognitive profiles in idiopathic normal pressure hydrocephalus and Alzheimer's disease by Magnhild S. Dejgaard, Per Kristian Eide, Gro Gujord Tangen, Eva Skovlund, Geir Selbæk and Torgeir Bruun Wyller in Journal of Alzheimer's Disease

sj-tif-2-alz-10.1177_13872877261478264 - Supplemental material for Cognitive profiles in idiopathic normal pressure hydrocephalus and Alzheimer's disease Supplemental material, sj-tif-2-alz-10.1177_13872877261478264 for Cognitive profiles in idiopathic normal pressure hydrocephalus and Alzheimer's disease by Magnhild S. Dejgaard, Per Kristian Eide, Gro Gujord Tangen, Eva Skovlund, Geir Selbæk and Torgeir Bruun Wyller in Journal of Alzheimer's Disease
