# Supplementary material for: Cognitive profiles in idiopathic normal pressure hydrocephalus and Alzheimer's disease
Source: J Alzheimers Dis. 2026 Aug 28;113(3):1458–66. doi: 10.1177/13872877261478264 (PMC13583080; doi:10.1177/13872877261478264)
Supplement: Supplementary file 1 [file sj-docx-1-alz-10.1177_13872877261478264.docx]

**S1, Supplemental Table 1:** Changes in reference interval for cerebrospinal fluid (CFS) amyloid – β (Aβ42) in the Norwegian Registry of patients assessed for cognitive symptoms (NorCog). Reference values are from the National user manual in Medical Biochemistry.

https://www.brukerhandboken.no/index.php?action=showtopic&book=biokjemi&topic=nsmstQau

| **Period** | **Reference interval** |
| --- | --- |
| Before June 2017 | ≥ 550 ng/L |
| 1 June 2017 - 1 October 2020 | ≥ 700 ng/L (Nunc-tubes) |
|  | ≥ 900 ng/L (Sarstedt-tubes) |
| 1 October 2020 - 30 June 2022 | > 1100 ng/L (Sarstedt-tubes) |
| From 1 July 2022 | > 1030 ng/L (Sarstedt-tubes) |

Nunc-tubes: used for CSF before March 2019. Sarstedt tubes were introduced in the NorCog biobank protocol from March 2019. New instrumentation (Roche Cobas e602) and updated cut-offs with a grey zone for Aβ42 (1000 – 1200 ng/L) improved diagnostic interpretation. Therefore, the cut-off was 1100 ng/L from 1. October 2020. From 1. July 2022 a new cut off at 1030 ng/L with an updated grey zone was established based on improved technology with better diagnostic reliability.

**S2, Supplemental Table 2.** Age range for normative populations not covering the entire age range of the study sample, number and age range of study patients aged outside that of the respective normative populations

| Cognitive test | Age range of normative populations (years) | iNPH | | | | AD | | | |
| --- | --- | --- | --- | --- | --- | --- | --- | --- | --- |
|  |  | Patients aged below normative population (n) | Lowest age (years) | Patients aged above normative population (n) | Highest age (years) | Patients aged below normative population (n) | Lowest age (years) | Patients aged above normative population (n) | Highest age (years) |
| RAVLT learning | 49 - 79 |  |  | 14 | 84 |  |  |  |  |
| CERAD Figure Construction Test | 60 - 79 | 3 | 52 | 28 | 84 | 68 | 49 | 38 | 89 |
| TMT A | 41 - 84 |  |  | 1 | 85 |  |  | 7 | 89 |
| TMT B | 41 - 84 |  |  | 1 | 85 |  |  | 1 | 85 |

iNPH = idiopathic normal pressure hydrocephalus, AD = Alzheimer’s disease. RAVLT = Rey Auditory Verbal Learning Test. CERAD = Consortium to Establish a Registry for Alzheimer’s disease, TMT = Trail Making Test

**S3, Supplementl Table 3** Number of patients who fulfilled the cognitive tests and number of patients with an available z-score. Due to missing information about years of education, z-scores were not available for all patients

| Cognitive tests | iNPH | | AD | |
| --- | --- | --- | --- | --- |
|  | n | z-scores available for (n) | n | z-scores available for (n) |
| CERAD 10-word immediate recall | 77 | 77 | 944 | 906 |
| CERAD 10-word delayed recall | 77 | 77 | 929 | 892 |
| RAVLT immediate recall | 199 | 199 | 21 | 19 |
| CERAD Figure Construction Test | 264 | 263 | 666 | 642 |
| TMT A | 269 | 273 | 1010 | 1028 |
| TMT B | 180 | 273 | 591 | 799 |
| Phonemic fluency | 246 | 244 | 870 | 844 |

iNPH = idiopathic normal pressure hydrocephalus, AD = Alzheimer’s disease. CERAD = Consortium to Establish a Registry for Alzheimer’s disease, RAVLT = Rey Auditory Verbal Learning Test. TMT = Trail Making Test

**S4, Supplemental Table 4**: Multinominal logistic regression analyses. For each cognitive test, the table shows the odds ratio (OR) for being severely impaired (z ≤ - 3) versus not impaired (z > -1.5) and for being mildly impaired (-3 < z ≤ -1.5) versus not impaired for patients with idiopathic normal pressure hydrocephalus (iNPH) compared to patients with Alzheimer’s disease (AD). The first column displays the main analysis, Clinical Dementia Rating Scale (CDR) category in three levels (CDR 0-0.5 = no or mild cognitive impairment, CDR 1 = mild dementia, CDR 2-3 = moderate or severe dementia) being included as a covariate. The second and third column are sensitivity analyses. Both displays crude analyses with only patient category (iNPH versus AD) as explanatory variable, based on all cases and cases restricted to those participating in the main analysis, respectively.

|  | **OR (95% CI) adjusted for CDR score** | | | **Crude OR (95% CI) All cases** | | | | **Crude OR (95% CI) restricted** | | |
| --- | --- | --- | --- | --- | --- | --- | --- | --- | --- | --- |
|  | **n** |  | **p-value** | **n** |  | **p-value** | | **n** |  | **p-value** |
| **Immediate recall** | 912 |  |  | 1201 |  |  | | 912 |  |  |
| Severe vs no impairment |  |  |  |  |  |  | |  |  |  |
| AD (ref) |  | 1 |  |  | 1 | |  |  | 1 |  |
| iNPH |  | 0.23 (0.14 to 0.37) | <0.001 |  | 0.25 (0.16 to 0.39) | | <0.001 |  | 0.22 (0.14 to 0.35) | <0.001 |
| Moderate vs no impairment |  |  |  |  |  | |  |  |  |  |
| AD (ref) |  | 1 |  |  | 1 | |  |  | 1 |  |
| iNPH |  | 0.43 (0.31 to 0.61) | <0.001 |  | 0.38 (0.28 to 0.52) | | <0.001 |  | 0.41 (0.29 to 0.57) | <0.001 |
| **Delayed recall** | 904 |  |  | 1184 |  | |  | 904 |  |  |
| Severe vs no impairment |  |  |  |  |  | |  |  |  |  |
| AD (ref) |  | 1 |  |  | 1 | |  |  | 1 |  |
| iNPH |  | 0.25 (0.16 to 0.41) | <0.001 |  | 0.22 (0.14 to 0.35) | | <0.001 |  | 0.24 (0.15 to 0.39) | <0.001 |
| Moderate vs no impairment |  |  |  |  |  | |  |  |  |  |
| AD (ref) |  | 1 |  |  | 1 | |  |  | 1 |  |
| iNPH |  | 0.57 (0.41 to 0.80) | <0.001 |  | 0.48 (0.35 to 0.65) | | <0.001 |  | 0.55 (0.40 to 0.76) | <0.001 |
| **Figure copying** | 698 |  |  | 905 |  | |  | 698 |  |  |
| Severe vs no impairment |  |  |  |  |  | |  |  |  |  |
| AD (ref) |  | 1 |  |  | 1 | |  |  | 1 |  |
| iNPH |  | 0.46 (0.31 to 0.70) | <0.001 |  | 0.47 (0.33 to 0.69) | | <0.001 |  | 0.46 (0.31 to 0.68) | <0.001 |
| Moderate vs no impairment |  |  |  |  |  | |  |  |  |  |
| AD (ref) |  | 1 |  |  | 1 | |  |  | 1 |  |
| iNPH |  | 0.75 (0.49 to 1.15) | 0.19 |  | 0.84 (0.56 to 1.26) | | 0.40 |  | 0.72 (0.47 to 1.11) | 0.14 |
| **TMT-A** | 971 |  |  | 1301 |  | |  | 971 |  |  |
| Severe vs no impairment |  |  |  |  |  | |  |  |  |  |
| AD (ref) |  | 1 |  |  | 1 | |  |  | 1 |  |
| iNPH |  | 0.23 (0.10 to 0.56) | <0.001 |  | 0.20 (0.09 to 0.47) | | <0.001 |  | 0.21 (0.09 to 0.50) | <0.001 |
| Moderate vs no impairment |  | to |  |  |  | |  |  |  |  |
| AD (ref) |  | 1 |  |  | 1 | |  |  | 1 |  |
| iNPH |  | 1.09 (0.80 to 1.48) | 0.60 |  | 1.05 (0.80 to 1.38) | | 0.71 |  | 0.96 (0.71 to 1.28) | 0.76 |
| **TMT-B** | 805 |  |  | 1072 |  | |  | 805 |  |  |
| Severe vs no impairment |  |  |  |  |  | |  |  |  |  |
| AD (ref) |  | 1 |  |  | 1 | |  |  | 1 |  |
| iNPH |  | 0.41 (0.23 to 0.75) | 0.004 |  | 0.34 (0.19 to 0.60) | | <0.001 |  | 0.37 (0.20 to 0.67) | 0.001 |
| Moderate vs no impairment |  |  |  |  |  | |  |  |  |  |
| AD (ref) |  | 1 |  |  | 1 | |  |  | 1 |  |
| iNPH |  | 1.06 (0.74 to 1.51) | 0.75 |  | 1.21 (0.88 to 1.66) | | 0.24 |  | 0.95 (0.68 to 1.34) | 0.77 |
| **Phonemic fluency** | 829 |  |  | 1088 |  | |  | 829 |  |  |
| Severe vs no impairment |  |  |  |  |  | |  |  |  |  |
| AD (ref) |  | 1 |  |  | 1 | |  |  | 1 |  |
| iNPH |  | 1.91 (1.04 to 3.51) | 0.037 |  | 1.29 (0.75 to 2.22) | | 0.36 |  | 1.63 (0.91 to 2.93) | 0.10 |
| Moderate vs no impairment |  |  |  |  |  | |  |  |  |  |
| AD (ref) |  | 1 |  |  | 1 | |  |  | 1 |  |
| iNPH |  | 1.43 (1.03 to 1.97) | 0.033 |  | 1.37 (1.01 to 1.84) | | 0.041 |  | 1.30 (0.94 to 1.78) | 0.11 |

TMT = Trail Making Test

**S5, Supplemental Table 5:** Z-scores for the cognitive tests by Clinical Dementia Rating Scale (CDR)**,** including missing CDR score as a separate category.

|  | **idiopathic normal pressure hydrocephalus** | | **Alzheimer’s disease** | |  |  |
| --- | --- | --- | --- | --- | --- | --- |
| **CDR** | **n** | **Median Z-score (IQR)** | **n** | **Median Z-score (IQR)** | **Median z-score differences, (95 % CI)*** | p-value** |
| **Immediate recall** | | | | | | |
| 0-0.5 | 189 | -1.64 (-2.33 to -0.84) | 384 | -2.19 (-2.77 to -1.67) | -0.47 (-0.67 to -0.29) | < 0.001 |
| 1 | 52 | -2.13 (-2.82 to -1.55) | 232 | -2.35 (-3.00 to -1.78) | -0.15 (-0.47 to 0.00) | 0.084 |
| 2-3 | 20 | -2.64 (-2.93 to -1.88) | 35 | -3.00 (-3.00 to -2.15) | -0.16 (-0.40 to 0.00) | 0.114 |
| Missing | 15 | -2.26 (-2.74 to -1.24) | 274 | -2.19 (-2.64 to -1.77) | -0.08 (-0.64 to 0.41) | 0.639 |
| **Delayed Recall** | | | | | | |
| 0-0.5 | 187 | -1.68 (-2.30 to -0.96) | 381 | -2.26 (-2.97 to -1.56) | -0.53 (-0.70 to -0.36) | < 0.001 |
| 1 | 52 | -2.09 (-2.71 to -1.39) | 230 | -2.32 (-3.00 to -1.63) | -0.17 (-0.40 to 0.00) | 0.100 |
| 2-3 | 20 | -2.17 (-3.00 to -1.61) | 34 | -2.77 (-3.00 to -1.94) | -0.04 (-0.73 to 0.09) | 0.353 |
| Missing | 14 | -1.71 (-2.20 to -0.88) | 266 | -2.34 (-2.92 to -1.87) | -0.71 (-1.27 to -0.18) | 0.004 |
| **Figure copying** | | | | | | |
| 0-0.5 | 185 | 0.20 (-1.40 to 0.40) | 277 | -0.50 (-2.50 to 0.40) | -0.05 (-0.20 to 0.00) | 0.139 |
| 1 | 47 | -1.50 (-3.00 to 0.20) | 153 | -2.30 (-3.00 to 0.30) | 0.00 (-0.50 to 0.00) | 0.576 |
| 2-3 | 18 | -2.65 (-3.00 to -0.50) | 18 | -3.00 (-3.00 to 0.30) | 0.00 (-0.50 to 0.40) | 0.616 |
| Missing | 13 | -0.50 (-3.00 to 0.40) | 194 | -0.50 (-3.00 to 0.40) | 0.00 (-0.20 to 0.70) | 0.938 |
| **TMT A** | | | | | | |
| 0-0.5 | 188 | -1.23 (-2.29 to -0.37) | 434 | -1.41 (-2.48 to -0.46) | -0.16 (-0.37 to 0.00) | 0.061 |
| 1 | 51 | -2.27 (-2.53 to -1.73) | 244 | -2.37 (-2.70 to -1.15) | -0.00 (-0.16 to 0.16) | 0.842 |
| 2-3 | 19 | -2.48 (-2.70 to -2.10) | 35 | -2.48 (-2.75 to -2.06) | 0.00 (-0.27 to 0.25) | 0.863 |
| Missing | 15 | -1.58 (-2.37 to -0.72) | 315 | -1.63 (-2.70 to -0.61) | -0.05 (-0.43 to 0.43) | 0.864 |
| **TMT B** | | | | | | |
| 0-0.5 | 188 | -2.07 (-2.58 to -0.85) | 370 | -2.32 (-2.69 to -1.21) | -0.17 (-0.32 to -0.03) | 0.013 |
| 1 | 51 | -2.41 (-2.61 to -2.17) | 157 | -2.44 (-2.84 to -2.14) | -0.01 (-0.16 to 0.11) | 0.767 |
| 2-3 | 19 | -2.45 (-2.62 to -2.27) | 20 | -2.53 (-2.76 to -2.34) | -0.07 (-0.30 to 0.15) | 0.482 |
| Missing | 15 | -2.37 (-2.76 to -2.07) | 252 | -2.40 (-2.97 to -1.14) | 0.06 (-0.13 to 0.37) | 0.811 |
| **Phonemic fluency** | | | | | | |
| 0-0.5 | 175 | -1.30 (-2.10 to -0.60) | 363 | -1.00 (-1.80 to -0.10) | 0.30 (0.10 to 0.50) | 0.003 |
| 1 | 43 | -2.20 (-2.70 to -1.40) | 201 | -1.50 (-2.20 to -0.80) | 0.50 (0.20 to 0.80) | 0.001 |
| 2-3 | 15 | -2.30 (-3.00 to -1.70) | 32 | -1.90 (-2.60 to -0.90) | 0.50 (0.00 to 1.10) | 0.068 |
| Missing | 11 | -1.90 (-2.40 to -0.80) | 248 | -1.25 (-2.10 to -0.20) | 0.50 (0.00 to 1.00) | 0.165 |

CDR; Clinical Dementia Rating Scale. IQR; Interquartile range. TMT; Trail Making Test. WMW: Wilcoxon Mann-Whitney test*Hodges-Lehmann estimator was used to analyze median z-score differences (AD – iNPH) group, ** Wilcoxon Mann-Whitney test was used to test the differences in cognitive test results.
